# *Lactobacillus plantarum* and Its Postbiotics Reverse Behavioral Deficits in a Fruit Fly Model of Alzheimer's Disease

**DOI:** 10.1002/alz70859_102447

**Published:** 2025-12-25

**Authors:** Yousef Addassi, Harrison Taylor, Alexa McDermott, Maddi Roberts, Brad Bennett, Patricia Jumbo‐Lucioni

**Affiliations:** ^1^ Samford University McWhorter School of Pharmacy, Birmingham, AL USA; ^2^ Samford University, Birmingham, AL USA; ^3^ Samford University Howard College of Arts and Sciences, Birmingham, AL USA; ^4^ University of Alabama at Birmingham College of Arts and Sciences, Birmingham, AL USA

## Abstract

**Background:**

Gut microbiota disruptions have been implicated in Alzheimer’s disease (AD) pathogenesis. *Lactobacillus* probiotics have demonstrated therapeutic potential in AD by modulating the gut microbiome, while *Lactobacillus* postbiotics, soluble factors secreted by live bacteria, confer similar effects with advantages like longer shelf life, lower cost, and reduced infection risk. Evidence of the benefits of postbiotics in AD is limited. This study compares the effectiveness of *Lactobacillus* probiotics and postbiotics in mitigating behavioral deficits in a Drosophila model of AD.

**Method:**

Flies overexpressing *human amyloid β precursor protein* and *β‐site APP cleaving enzyme* in neurons served as AD model. *Lactobacillus plantarum (Lp)* was prepared at 1.0 x 10⁹ CFU/µL in MRS Lactobacilli broth, with the upper 80% of culture supernatant filtered as postbiotic fraction (*Lp*‐PBx). *Lp* and *Lp*‐PBx were diluted 1:2 in 5% sucrose and administered via capillaries in four 24‐hour doses over two weeks. Food intake was recorded. Locomotion and memory were assessed at 14 days. Locomotion was evaluated using a negative geotaxis assay testing flies' ability to cross 8‐cm in 10 seconds. Memory was tested through an aversive phototaxic suppression assay, where flies were trained to associate light with an aversive odor. After ten training cycles, flies were tested for dark preference. Data was stratified by sex and analyzed through a two‐way ANOVA to test the main effects of genotype, treatment, and their interaction.

**Result:**

Untreated AD males (0.48±0.03 vs 0.80±0.03, *p*<0.0001) and females (0.39±0.04 vs 0.70±0.04, *p*<0.0001) exhibited significant mobility impairments compared to controls. However, AD flies fed *Lp* or *Lp*‐PBx showed restored mobility, climbing at speeds comparable to controls. Specifically, AD males fed *Lp* (0.73±0.03) or *Lp*‐PBx (0.75±0.03) and females fed *Lp* (0.61±0.04) or *Lp*‐PBx (0.59±0.04) climbed significantly faster than untreated counterparts (males: 0.48±0.03, *p*<0.0001; females: 0.39±0.04, *p*<0.05). While food intake in females was unaffected, males fed *Lp* ate significantly less than those given *Lp*‐PBx (*p*=0.0216), regardless of genotype. Memory assays are ongoing.

**Conclusion:**

In summary, our findings suggest *Lp*‐PBx as a viable alternative to *Lp* for managing mobility deficits in a Drosophila model of AD. Its palatability facilitates administration, making it a practical therapeutic option.

